# Influence of Coarse Cement Particle Content on Intrinsic Self-Healing of Mortar

**DOI:** 10.3390/ma18102216

**Published:** 2025-05-11

**Authors:** Xingkong Ma, Wu Yao, Anming She, Yongqi Wei

**Affiliations:** Key Laboratory of Advanced Civil Engineering Materials of Ministry of Education, School of Materials Science and Engineering, Tongji University, Shanghai 201804, China; 2230650@tongji.edu.cn (X.M.); sheanming@tongji.edu.cn (A.S.); yq.wei@tongji.edu.cn (Y.W.)

**Keywords:** self-healing, particle size distribution, ultrasonic pulse velocity, hydrated encapsulation shell

## Abstract

This study investigated the effect of coarse cement on the self-healing ability of mortar. Coarse cement, prepared via negative-pressure screening, was substituted (0–40%) in mortar mixes with water/cement (w/c) ratios of 0.45–0.55. The specimens were cured for 28 days, cracked, and allowed to self-heal for another 28 days. Self-healing was evaluated based on compressive strength recovery and ultrasonic pulse velocity. At a 0.50 w/c ratio, 10% coarse cement substitution achieved 87.7% strength recovery (21.2 MPa increase), outperforming the control group (74.1%, 13.0 MPa). Reducing the w/c ratio to 0.45 further enhanced recovery to 89.4% (21.5 MPa). While coarse particles alone reduced the initial strength, combining their addition (e.g., 10%) with a lower w/c ratio (0.45) improved self-healing without significant strength loss. Based on the Krstulović–Dabić model and a micro-geometric model incorporating hydration units, this study analyzed the intrinsic self-healing mechanism of cement-based materials through computational results. Theoretical calculations demonstrated that during cement hydration, coarser particles can form a microcapsule-like structure where hydration products encapsulate unhydrated cement. The findings suggest that optimizing coarse particle content and the w/c ratio can balance self-healing enhancement and mechanical performance, offering a viable strategy for energy saving and emission reduction by reducing the carbon emissions per unit of service life and the grinding process in cement production.

## 1. Introduction

Self-healing cementitious materials refer to those materials capable of autonomously repairing microcracks, without requiring external detection systems or human intervention [1]. Upon damage initiation within or on the material’s surface, the embedded repair agents are autonomously activated to restore the substrate, effectively sealing microcracks while recovering both mechanical integrity and long-term durability. Common self-healing cement-based materials often require the addition of various engineered additives to the cement substrate to achieve self-healing performance, such as microorganisms [2,3,4], hollow fibers [5,6,7], microcapsules [8,9], or shape memory alloys (SMA) [10,11,12]. In addition, cement-based materials generally have a certain self-healing ability. The intrinsic self-healing capacity of cementitious materials can be enhanced through the incorporation of supplementary cementitious materials (SCMs), such as fly ash, limestone powder, and granulated blast furnace slag [13,14,15].

There are different explanations for the intrinsic self-healing mechanism of cement-based materials. Sahmaran et al. [16] found that, compared with fly ash, slag has more positive effects on self-healing, which may be caused by the higher pH of slag pore solution and the higher levels of calcium oxide, which is beneficial to the precipitation of calcite. Nataliya et al. [17] clarified the difference between the self-sealing effect, the autogenous healing effect, and the continuing hydration process of cementitious materials. It is generally recognized that the self-healing of cementitious materials is due to complex reasons, including both chemical processes and physical processes [18]. The intrinsic self-healing mechanism of cementitious materials includes (a) the carbonization of calcium hydroxide, (b) the crack being filled by particles falling off the fracture surface or other substances in water, (c) the expansion of the hydration cementitious materials matrix on both sides of the crack, and (d) cement particles continuing to hydrate. Among these mechanisms, the carbonation of calcium hydroxide plays a leading role [19]. Nishiwaki et al. [20] provided a strong argument for this conclusion from the micro and macro perspectives: calcium carbonate crystals were observed at the cracks of self-healing cementitious material. Self-healing in early cementitious materials is chiefly caused by the further hydration of unhydrated cement, and the formation of calcium carbonate is the most probable cause of the self-healing effect in long-aged cementitious materials. Although the self-healing phenomenon caused by the continuous hydration of cement is often more pronounced at the early stage of hydrating cementitious material, it is still possible to maintain this self-healing ability at the later stage of hydration [21]. Suleiman and Nehdi [22] discovered that the environmental conditions during the healing process significantly affect both the self-healing products and the self-healing effectiveness of cementitious material. Tomczak et al. [23] showed that even though the self-healing ability of concrete after 20 months is decidedly weaker than that after 2 months, it still has a low water/cement ratio, and a lower water/cement ratio might have a more visible healing outcome. Jacobsen et al. [24] and Aldea et al. [25] found that in the detection of self-healing efficiency, the results obtained with the two methods of compressive strength recovery rate are different from those obtained with the ultrasonic method. Rahmani et al. [26] pointed out that the self-healing ability of concrete was better with coarser cement particles as a repair agent. Research by Yuan et al. [27] indicated that coarse-grained cement particles (30–60 μm) may play a key role in the intrinsic self-healing of cementitious materials. Lv and Chen [28,29,30] established a micro-damage model and analyzed the role of coarse cement particles in self-healing as well as their self-healing potential. Li et al. [31] pointed out that with the same coarse cement content, when the damage level exceeds the threshold, the self-healing rate of concrete decreases with an increasing degree of damage but increases with larger coarse cement particle diameters. However, when the damage level is below the threshold, the self-healing rate increases with both higher damage levels and larger coarse cement particle diameters. Wu et al. [32] believed that, compared with traditional cementitious materials, engineering cementitious materials have higher cementitious component content and a lower water/cement ratio, that their excellent crack width control ability gives them higher self-healing potential, and that more effort should be devoted to manufacturing intelligent engineering cementitious materials with effective and reliable self-healing properties. Liu et al. [33] found that cement with a low strength grade has more unhydrated parts in concrete specimens of the same age, which is caused by a higher content of coarse particles, and the self-healing effect is better than concrete made from cement with a high strength grade.

However, cement particles that are incompletely hydrated and encapsulated by hydration products can be regarded as self-healing microcapsules, with hydration products as the capsule shell material and unhydrated cement as the core material. Increasing the content of coarse particles by optimizing the particle size distribution of cement is equivalent to incorporating microcapsules into the cement substrate. With the formation of cracks in the cement substrate, the outside hydration product shell of the cement microcapsules at the crack site peels off with the crack, and the unhydrated cement particles are exposed to the external environment, which provides conditions for the rehydration reaction, generating new hydration products to fill the cracks, and causing the cement substrate to self-heal. This self-healing cementitious material, based on microcapsules composed of cement particles encapsulated by hydration products, can increase the service life to reduce carbon emissions per unit of service life by spontaneously repairing cracks in the substrate; however, this should be weighed against possible increases in cement consumption in new structures.

Moreover, in the cement production process, finer cement requires more surface energy than coarser cement with the same mass, which means more energy consumption during the grinding process. Therefore, by optimizing the particle size distribution of cement and increasing the content of coarse particles, energy consumption can be reduced, which also means reducing the carbon emissions per unit mass of the cementitious material production process.

In traditional self-healing techniques, polymer materials are often used as the capsule wall materials for self-healing microcapsules, which requires additional materials. The preparation process of these additional materials also generates carbon emissions. Moreover, to apply these technologies to the actual production or construction of cementitious materials, further steps and processes are involved, which also generate extra energy consumption and carbon emissions. Therefore, compared to traditional microcapsule self-healing technology, directly using coarse cement particles encapsulated by hydration products as microcapsules only requires optimizing the particle size distribution of cement, which is simpler, more economical, and environmentally friendly.

This innovation does not require additional consideration of the compatibility and chemical stability issues between microcapsules and cement in terms of performance, as the capsule wall material is the hydration product itself, which does not become a weak point in the cement substrate or lead to a significant decrease in strength.

The purpose of this study was to investigate the effect of cement containing coarse particles on the self-healing and mechanical properties of mortar-hardened paste and to analyze the reasons for this effect from a theoretical perspective. A compressive strength trial was used to measure the self-healing effect and mechanical properties, while ultrasonic pulse velocity (UPV) testing and photography played auxiliary roles in characterization. This work is expected to provide new ideas for optimizing cement particle size distribution and reducing energy consumption and carbon emissions.

## 2. Experimental Program

### 2.1. Materials

**Figure 1:**
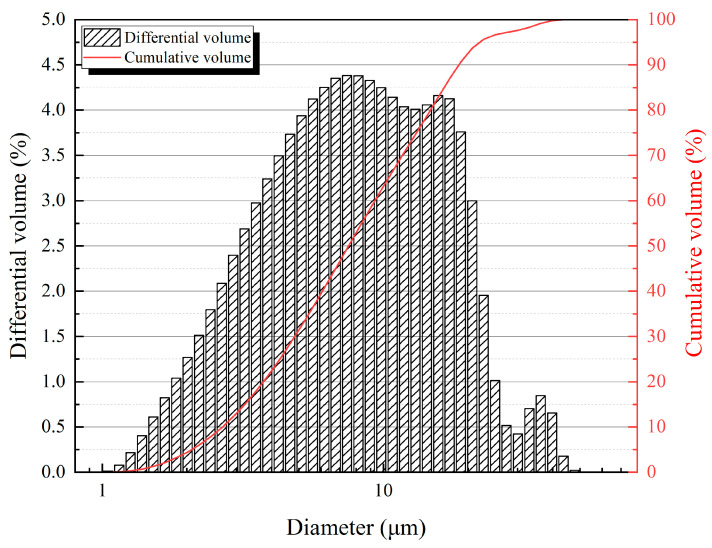
Particle size distribution (volume) of P.I. 42.5 cement.

ISO standard sand [34] was used in this study as the fine aggregate. The cement used was P.I. 42.5 cement from Shandong Province, China. The particle size distribution, measured using an LS 13320 laser diffraction (Beckman Coulter, Inc., Brea, CA, USA), is shown in Figure 1.

A cement fineness negative-pressure sieve analyzer was utilized to isolate coarse cement fractions specifically for subsequent experiments. The aperture of the sieve used to segregate cement was 45 μm. To ensure the uniformity of the product, the negative pressure should be controlled at 4.5 ± 0.3 kPa, and the mass of cement for each segregation should be controlled at 20.0 ± 0.5 g. To ascertain the appropriate screening time based on the size distribution of the cement particles, an LS 13320 laser diffraction was employed.

**Figure 2:**
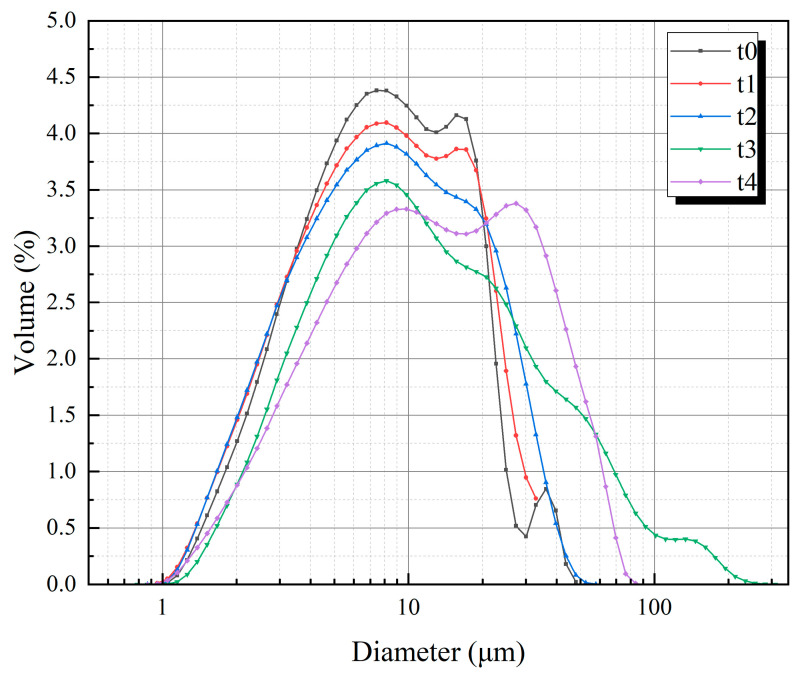
Size distribution of cement obtained with different screening times.

The size distribution and the median particle size of the cement obtained with different screening times are shown in Figure 2 and Table 1. The increase in screening time leads to an increase in the median particle size, and the proportion of finer particles presents a decreasing trend. When the screening time reaches 120 s, the median particle size increases relatively significantly. Thus, the time of each screening is determined to be 120 s, and the cement containing coarse particles is prepared in this way.

**Figure 3:**
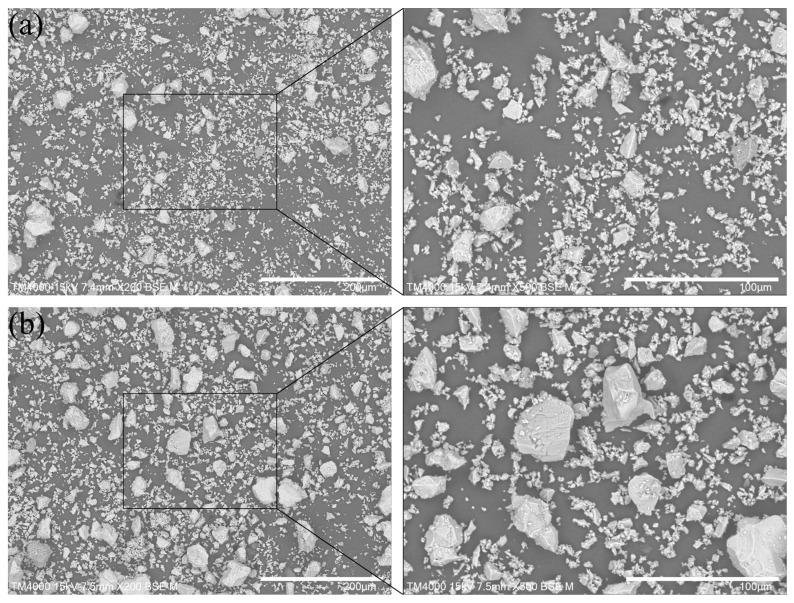
Images of cement particles (**a**) before screening, and (**b**) after screening.

**Figure 4:**
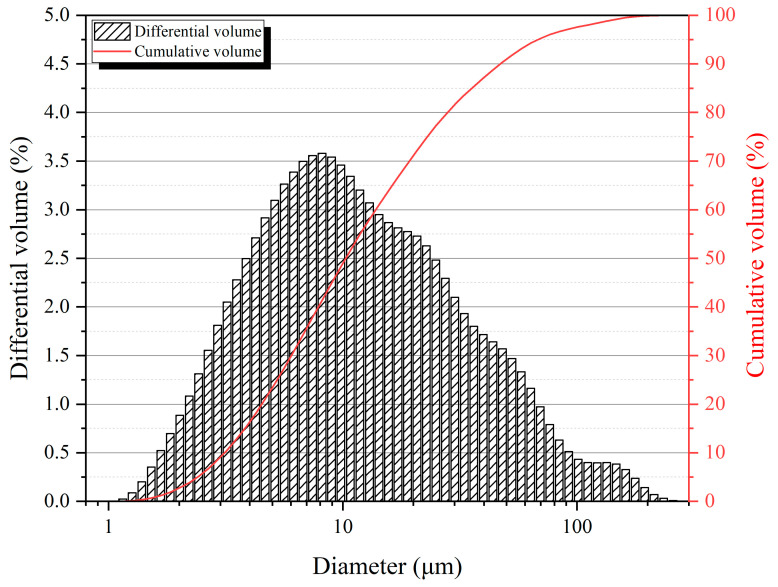
Particle size distribution (volume) of cement containing coarser particles.

Photographs of the cement, shown in Figure 3, were obtained using SEM (TM 4000, HITACHI, Tokyo, Japan). The content increase in coarse particles in the cement is apparent, but there are many smaller particles adsorbed onto the particles with larger diameters. The particle size distribution of the cement used as cement containing coarse particles in this study is shown in Figure 4. It is obvious that the content of coarse particles with a size of more than 45 μm increased after screening.

### 2.2. Specimen Preparation

To investigate the effect of cement containing coarse particles on self-healing and mechanical properties, five groups of mortar with different proportions of cement were designed. The detailed mixture proportions are given in Table 2. The mortar was mixed using the method in GB/T17671-2021 [34]. Cubic mortar specimens of 40 mm × 40 mm × 40 mm were prepared for compressive strength and ultrasonic pulse velocity characterizations.

### 2.3. Testing

**Figure 5:**
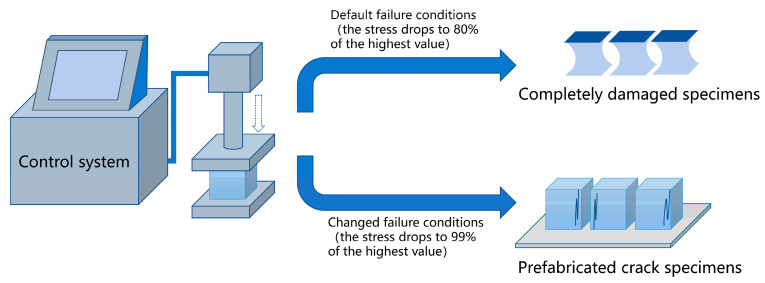
Schematic diagram of the prefabricated crack manufacturing process.

The test method refers to the standard GB/T17671-2021. After curing for 28 days, half of the specimens were used to prepare pre-damaged specimens. The specimens were placed on the test bench of the pressure testing machine and loaded at a rate of 2.4 kN/s. To obtain pre-damaged specimens that retained a certain level of integrity, we adjusted the parameters within the automatic control program to determine the failure conditions of the specimens, specifically when the load exceeded 20 kN and the strength of the specimens dropped to 99% of its initial value (the primary aim of ensuring that the loading force exceeded 20 kN was to prevent the loading process from halting due to minor disturbances experienced by the sensor during the initial loading stage). A schematic diagram of the method for prefabricating cracks in the specimens is shown in Figure 5.

**Figure 6:**
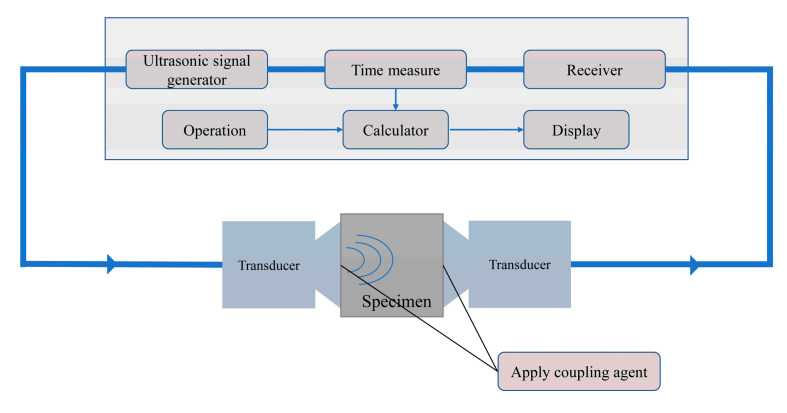
Schematic diagram of the UPV tests.

At the age of 28 days after standard curing, the ultrasonic velocity of the specimens of the a and b series was tested by using the ultrasonic instrument (ZBL-U250, Beijing ZBL Science And Technology Co., Ltd., Beijing, China). The test method refers to the standard [35]. During the testing process, white petroleum jelly was used as a coupling agent. At the age of 56 days after standard curing (the healing age of the pre-damaged specimens was 28 days), the ultrasonic velocity of the c and d series was tested. The two transducers of the instrument were placed on opposite sides of the specimen. When testing the specimens with prefabricated cracks, a specific test direction had to be selected so that the propagation path of the ultrasonic wave in the specimens passed through the main cracks of the pre-damaged specimens. At the age of 28 days after standard curing, the compressive strength of the specimens of series a and the compressive strength of the pre-damaged crack specimens of series b were tested with a pressure testing machine. The specimens of series d and the prefabricated crack specimens of series c continued to undergo standard curing for 28 days. After the curing was completed, the compressive strength of series c and d was tested using an industrial camera (Haiying Feiteng Technology Co., Ltd., Shenzhen, China) to photograph the healing of the prefabricated crack on the surface of specimens during the 28-day healing process. A schematic diagram of the method for UPV tests is shown in Figure 6.

**Figure 7:**
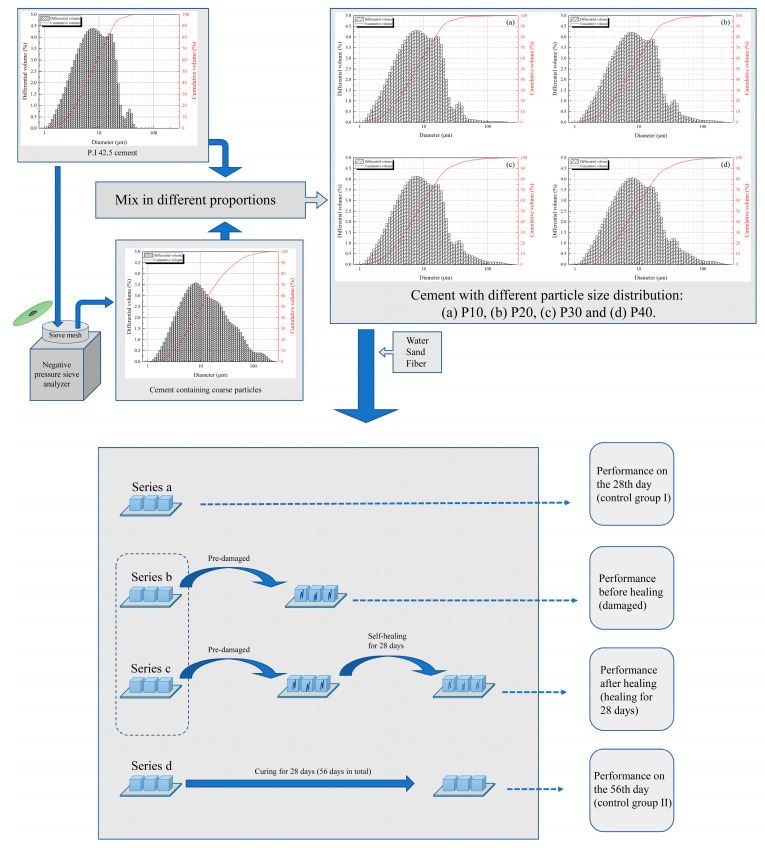
Schematic diagram of the experiment.

In this study, the compressive recovery ratio was defined as the ratio of the compressive strength value after self-healing to that of uncracked specimens at the same curing age, specifically representing the compressive strength ratio between series c and series d specimens. Furthermore, the ultrasonic velocity increase rate was defined as the ratio of the increment in ultrasonic pulse velocity (UPV) before and after self-healing to the UPV value measured in pre-cracked specimens prior to self-healing. A schematic diagram of the experiment is shown in Figure 7.

## 3. Results and Discussion

### 3.1. Crack Recovery

**Figure 8:**
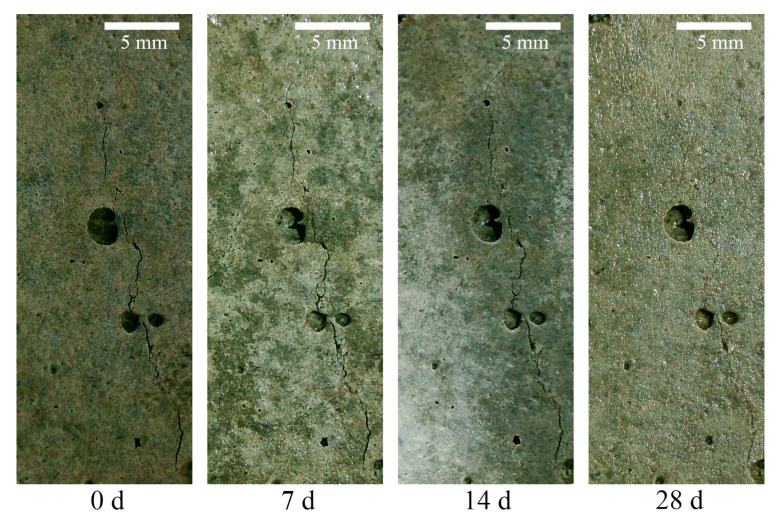
Images of crack recovery (P10-0.50 group).

During the healing for 28 days of the pre-damaged specimens, the crack image changes are as shown in Figure 8. It can be seen that, with an increase in the healing age, some cracks almost completely closed after 28 days of healing. It can be inferred that some small cracks inside the specimens also closed to a certain extent, which could explain the improved mechanical properties of the pattern at the macrostructural level.

### 3.2. Compressive Strength

**Figure 9:**
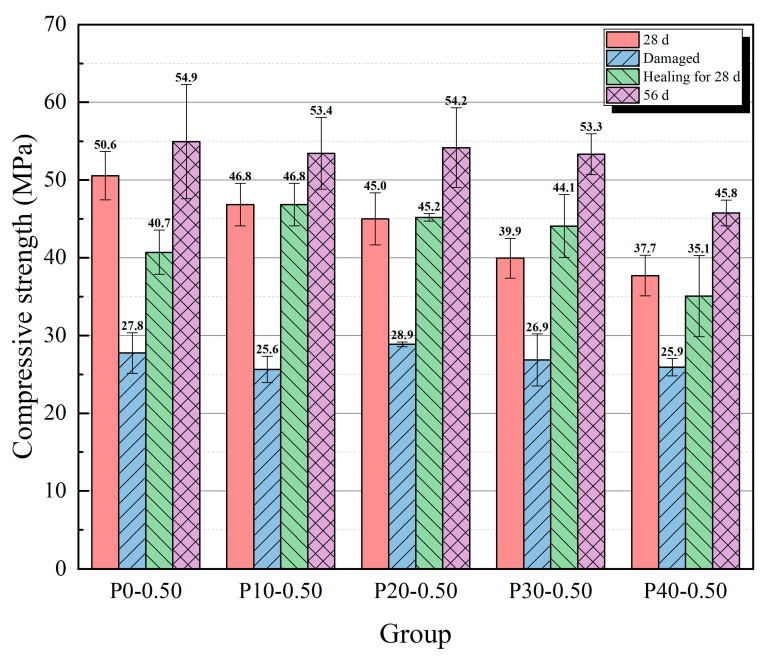
Results of compressive strength test with 0.50 water/cement ratio.

**Figure 10:**
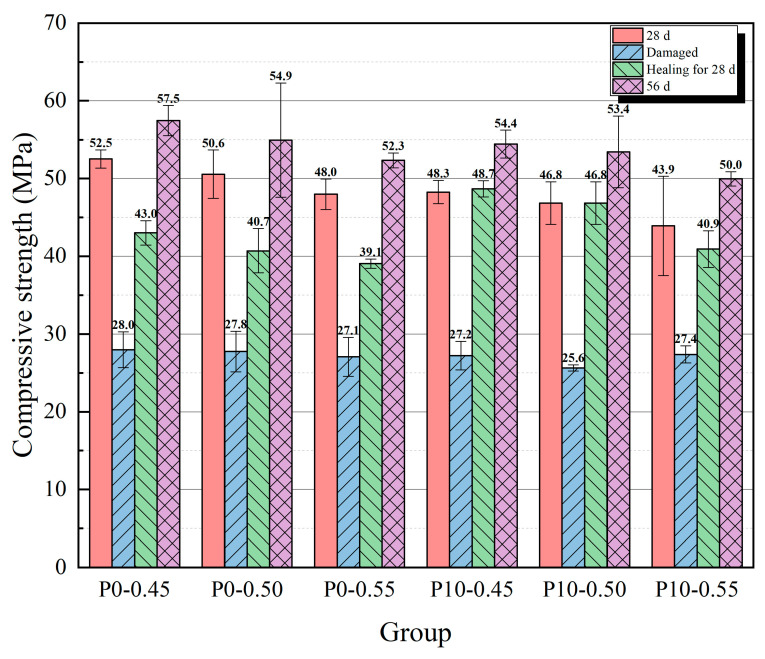
Results of compressive strength tests with different water/cement ratios.

The results of the compressive strength tests are shown in Figure 9 and Figure 10, and the compressive strength recovery rates of each group are presented in Table 3. With an increase in the content of cement containing coarse particles, the strength at 28 days showed a downward trend because the mortar with a higher content of coarse cement particles had a lower degree of hydration. A lower degree of hydration makes it difficult for hydration products to fill the spaces within the mortar, leading to an increase in porosity, which subsequently affects its strength development. In contrast, the strength at the age of 56 days of the three groups with higher coarse particle content (P10-0.50, P20-0.50, and P30-0.50) was not significantly lower than that of the P0-0.50 group. The reason for this phenomenon may be that, as the age increased, the group containing coarser particles exhibited a higher hydration rate for some time, while the group without coarse particles had already essentially completed hydration, with little change in the degree of hydration. When the content of coarse particle cement reached 40%, the strength at the age of 56 days decreased significantly. The compressive strength of the specimens at the age of 28 days was greatly reduced, to about 27 MPa after the pre-damaged step.

After healing for 28 days (56 days after the specimens were formed), the compressive strength of all groups of specimens had recovered to a certain extent. Among them, the compressive strength of the P10-0.50 and P20-0.50 groups was basically the same as the strength at the age of 28 days, and the strength of the P30-0.50 group was even slightly higher than its 28-day strength. The compressive strength of the P40-0.50 group had not recovered to its strength before the destruction at 28 days. Among groups with a 0.50 water/cement ratio, the P10-0.50 group had the highest recovery of compressive strength (87.66%), while the recovery of compressive strength of the P0-0.50 group was 74.11%. The recovery rate was lower than that observed in the experiments conducted by Wang et al. [36] based on a 7-day cracking age (approximately 93%). This discrepancy may be attributed to the reduced content of unhydrated cement within the mortar at the 28-day cracking age. The 28-day and 56-day compressive strengths of the P10-0.50 group did not show a significant decline, reaching 92.65% and 97.28% of those in the P0-0.50 group, respectively.

The strength at 28 days increased with a decrease in the water/cement ratio, and the reason is that a considerable portion of the pore volume is caused by unreacted water; a lower water/cement ratio often means a lower porosity. After healing for 28 days, the P10 groups showed a better strength recovery than the P0 groups; the strength of all the P10 groups was close to, or even slightly exceeded, their strength at the age of 28 days. Between groups with the same coarse particle content but a different water/cement ratio, the recovery of compressive strength showed an upward trend as the water/cement ratio decreased. This result may be due to the effect of a higher water/cement ratio, which decreased the compressive strength at the age of 56 days. Regarding the effect of the water/cement ratio on strength recovery, after healing for 28 days, the strength of the P10-0.55 group was closer to its strength at the age of 56 days. However, this is more likely because the higher water/cement ratio significantly reduced strength at the age of 56 days; thus, the absolute increase in strength during the healing process is an important indicator for evaluating the recovery ability. The absolute increase in strength shows a decreasing trend with an increase in the water/cement ratio. Based on the above results, the P10-0.45 group achieved the best balance between compressive strength and self-healing ability, as the 28-day and 56-day compressive strength values of P10-0.50 reached 95.45% and 99.09% of those in the P0-0.50 group, respectively. Additionally, the recovery rate of P10-0.45 reached 89.44%.

### 3.3. Ultrasonic Pulse Velocity

**Figure 11:**
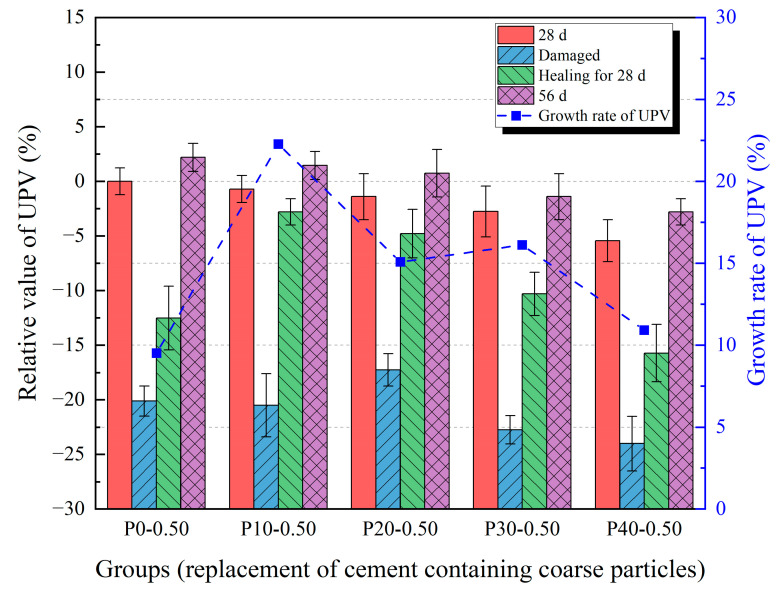
Results of UPV test with 0.50 water/cement ratio.

**Figure 12:**
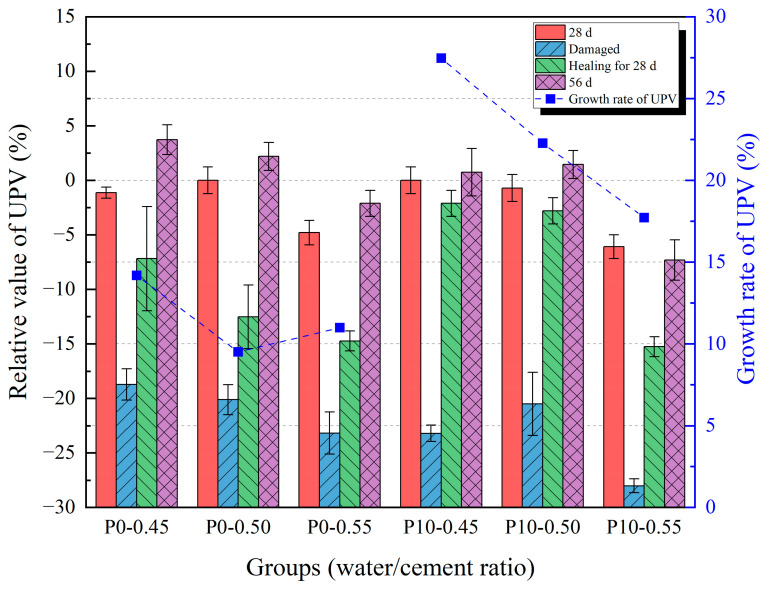
Results of UPV test with different water/cement ratios.

The results of the UPV testing are shown in Figure 11 and Figure 12. Relative values were used to normalize the results and minimize the influence of specimen variability and geometric effects due to the non-standard size. The ultrasonic velocity of the damaged specimens was significantly lower than the velocity of the undamaged specimens. This is because, in the pre-damaged specimens with cracks, the path of ultrasonic wave propagation in the solid was cut off by air, so the path of acoustic wave propagation increased, resulting in a decrease in sound velocity. The ultrasonic velocity of the specimens at 56 days was slightly larger than the wave velocity at the same age. This is because the cement in structurally complete specimens continued to hydrate, improving the compactness of the specimen, but its influence on the acoustic wave propagation path was less significant than the influence of cracks on the propagation path. After healing for 28 days, the ultrasonic wave velocity of the damaged specimens had significantly improved. This is because after some microcracks in the specimens were healed, the acoustic wave had a shorter propagation path compared to when it was just destroyed. Compared with the results of compressive strength, the results of the UPV test of the specimens after healing are not the same as those of the specimens before destruction, which may be due to the large cracks that still exist after the healing of the specimens. A similar phenomenon was observed in the study by Wang et al. [37]. After 28 days of self-healing, the ultrasonic wave velocity recovered to approximately 67% of its pre-damage level. Even if the width and depth of these large cracks are reduced, the path of acoustic wave propagation cannot be restored to the nearly linear propagation path in the undamaged state. Therefore, although the compressive strength of the two series of specimens is similar, the ultrasonic velocity is still low. It is worth mentioning that the increasing trend of healing ability with a higher water/cement ratio is clearer in the growth rate of UPV.

**Figure 13:**
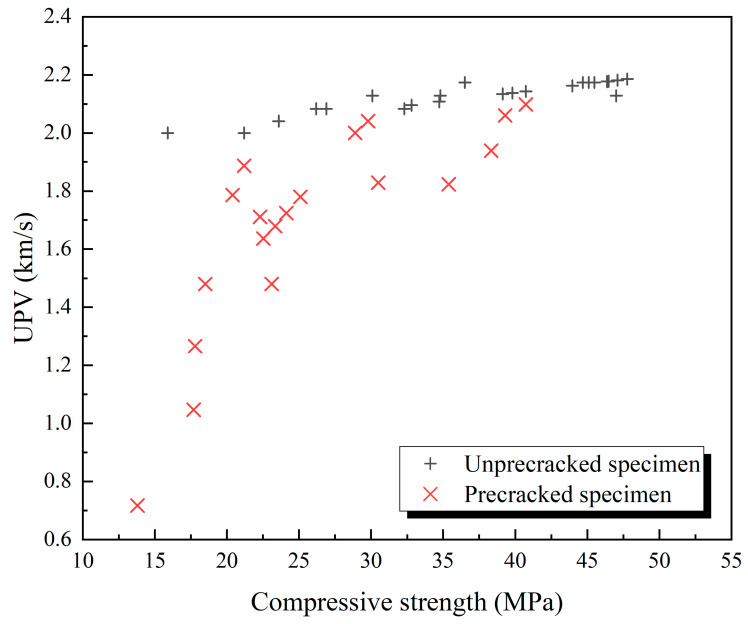
Correlation comparison between ultrasonic velocity and compressive strength: independently prepared prefabricated crack specimens vs. intact specimens.

To investigate the distinction in the relationship between ultrasonic velocity and compressive strength of specimens with prefabricated cracks compared with undamaged specimens, a newly prepared independent set of specimens with systematically varied strengths was fabricated. These specimens, specifically designed as a separate experimental group, had their compressive strength controlled throughout different curing ages. The comparative results between the freshly fabricated crack-bearing specimens and intact specimens are presented in Figure 13. The experimental results show that the ultrasonic velocity of the specimens destroyed by the pressure testing machine is significantly different from that of the undamaged specimens. Even if the destroyed specimens and the undamaged specimens have similar compressive strength values at an early age, the ultrasonic velocity of the destroyed specimens is still significantly lower than that of the undamaged specimens. In addition, the ultrasonic velocity of the undamaged specimens and the prefabricated crack specimens increases with compressive strength, but the ultrasonic velocity of the former increases only slightly, whereas that of the latter increases significantly when the compressive strength is low and slows down when the compressive strength is high. In addition, compared with the undamaged specimens, the data of the prefabricated crack specimens have a larger discrete value, which is likely due to the existence of large cracks, and the crack shape of the different specimens varies, demonstrating that there is a large difference in the change of the path of sound wave propagation in the specimens.

### 3.4. Theoretical Analysis and Calculation

According to the model proposed by Krstolovic et al. [38], cement is simultaneously affected by three dynamic factors at different stages of hydration: nucleus and crystal growth (NG), interactions at phase boundaries (I), and diffusion (D). However, the dominant factors vary at different stages. The cement hydration rate $\left[d\alpha /dt\right]$ during these three stages satisfies the following three equations:

(1) $${\left[d\alpha /dt\right]}_{NG}=K_{NG}n\left(1-\alpha \right){\left[-\ln\left(1-\alpha \right)\right]}^{1-1/n}$$

(2) $${\left[d\alpha /dt\right]}_{I}=3K_{I}\sqrt[{3}]{{\left(1-\alpha \right)}^{2}}$$

(3) $${\left[d\alpha /dt\right]}_{D}=3K_{D}\sqrt[{3}]{{\left(1-\alpha \right)}^{2}}/2(1-\sqrt[{3}]{1-\alpha })$$

where $K_{NG}$ is the rate constant of the crystallization nucleation reaction, $K_{I}$ is the rate constant for the reaction at phase boundaries, $K_{D}$ is the diffusion process rate, and n is a constant related to the mineral composition of cement, which describes the geometric growth of crystals.

**Figure 14:**
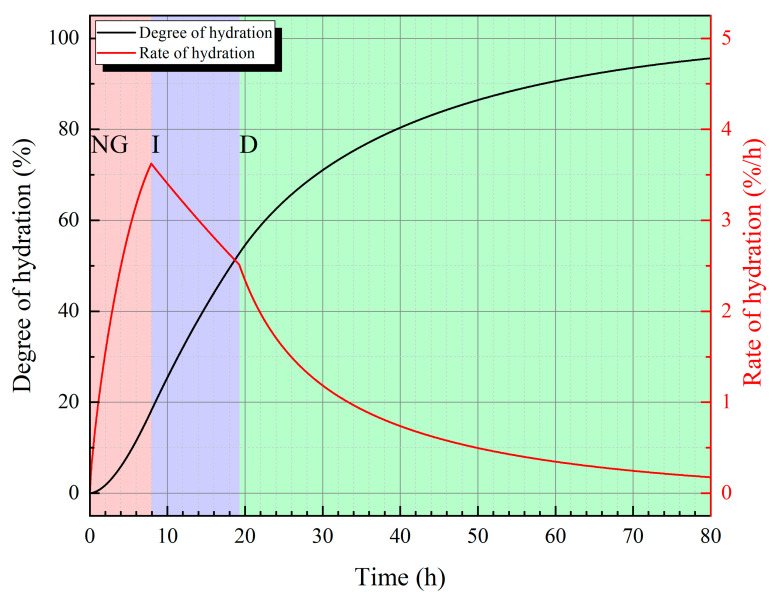
Relationship between degree and rate of hydration and time.

The hydration kinetics parameters of cement are shown in Table 4, and the calculated results are shown in Figure 14. At the early stage of hydration, the hydration rate is mainly controlled by crystallization nucleation reaction; this stage usually lasts for several hours. In the next few hours, the reaction at phase boundaries becomes the dominant factor. The third stage begins at about 20 h, with the diffusion process as the main factor.

**Figure 15:**
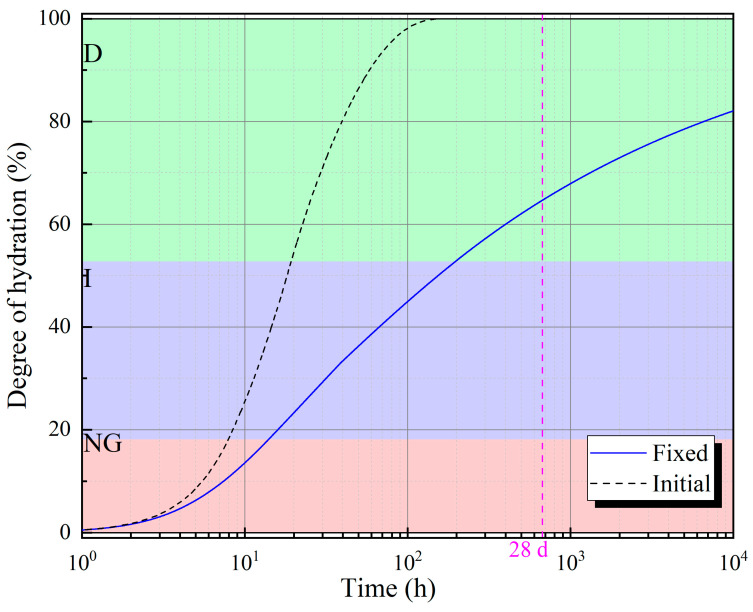
Relationship between fixed degree of hydration and time.

However, this model does not consider the effect of the residual area of cement particles on the reaction rate during the hydration process of these particles. With hydration, cement particles will no longer be isolated, and hydration products make them interconnected. Therefore, for a single cement particle, the area that can be used for reaction (the area in contact with water) is reduced. Jin et al. [39] revised Krstulović’s formula by using a series of conversion coefficients, and their simulation results have a good fitting effect over a longer hydration age. The simulation results obtained using their method and the parameters used in this study are shown in Figure 15. The overall hydration degree of cement at the age of 28 days is about 66.6%.

The above hydration degree is based on a single particle size. In fact, when the hydration depth of cement particles of different sizes is the same, their hydration degree is not the same. We can assume that, during the hydration process of cement particles, the dissolution and hydration depth of cement particles of different sizes are independent of the size of the particles themselves; that is, at the same age, t, they have the same hydration depth, h(t).

The consumed volume during the hydration process of a single particle satisfies the following formula:

(4) $$dV\left(R\right)=\left\{\begin{array}{c} k_{s}d\left(R^{3}\right),{\;\;\;\;\;\;\;\;\;\;\;\;\;\;\;\;\;\;\;\;\;\;\;\;\;\;\;\;\;R}_{min}\leq R\leq h\left(t\right)\\ k_{s}d\left(R^{3}-{\left(R-h\left(t\right)\right)}^{3}\right),\;\;\;h\left(t\right)<R\leq R_{max} \end{array}\right.$$

where R is the initial radius of cement particles, $k_{s}$ is the shape factor, and when cement particles are considered spherical, $k_{s}=4\pi /3$.

According to the particle size distribution of cement, its overall hydration volume meets the following requirements:

(5) $$V_{h}\left(t\right)=\int_{R_{min}}^{h\left(t\right)}k_{s}d\left(n\left(R\right)\cdot R^{3}\right)+\int_{h\left(t\right)}^{R_{max}}k_{s}d\left(n\left(R\right)\cdot \left(R^{3}-{\left(R-h\left(t\right)\right)}^{3}\right)\right)$$

or

(6) $$V_{h}\left(t\right)=\int_{R_{min}}^{h\left(t\right)}k_{s}dv\left(R\right)+\int_{h\left(t\right)}^{R_{max}}kd\left((R^{3}-{\left(R-h(t)\right)}^{3}\;)v(R)/R^{3}\right)$$

where n (R) represents the relationship between the particle radius distribution and particle radius of cement based on quantity representation, and v (R) represents the relationship between the particle radius distribution and particle radius of cement based on volume representation. Since the particle radius distribution is given in proportion, the numerical integration result is the proportion of the hydration volume, equivalent to the overall hydration degree.

**Figure 16:**
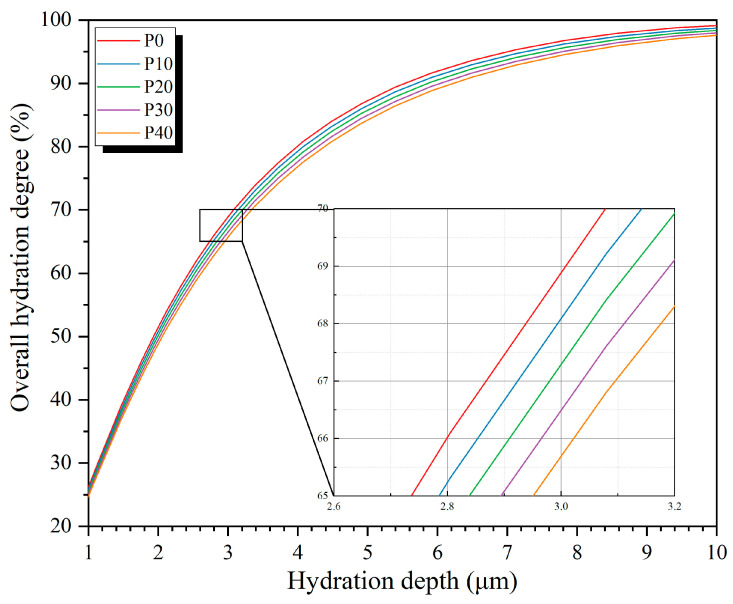
Relationship between overall hydration degree and hydration depth.

**Figure 17:**
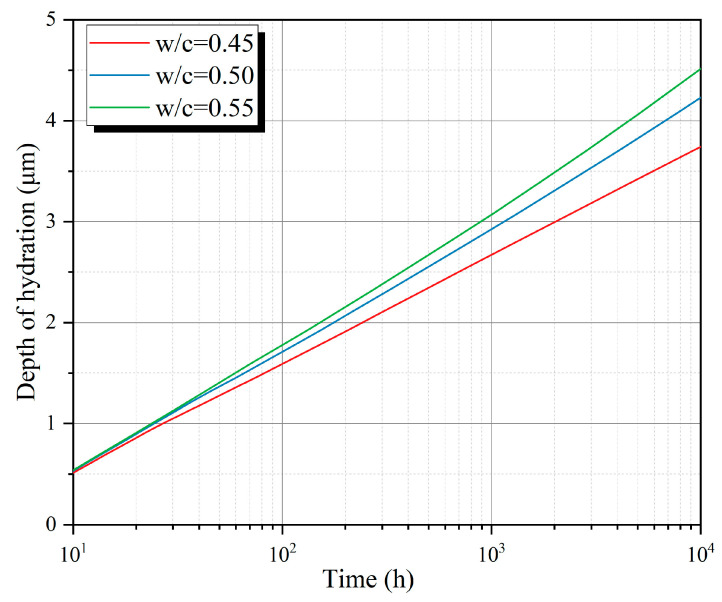
Relationship between depth of hydration and hydration time.

It is then necessary to establish the relationship between  h(t)  and $V_{h}(t)$ based on Formulas (4)–(6), that is, the relationship between the hydration depth of particles and the overall hydration mass. By substituting the particle size distribution into the above formula, the relationship between overall hydration degree and hydration depth can be obtained, as shown in Figure 16. The overall hydration degree of the P0-0.50 group at the age of 28 days is 66.6%, and by substituting this value into the above formula, the hydration depth is 2.81 μm. The relationship between depth of hydration and hydration time is shown in Figure 17 (groups P0-0.45, P0-0.50, and P0-0.55).

**Figure 18:**
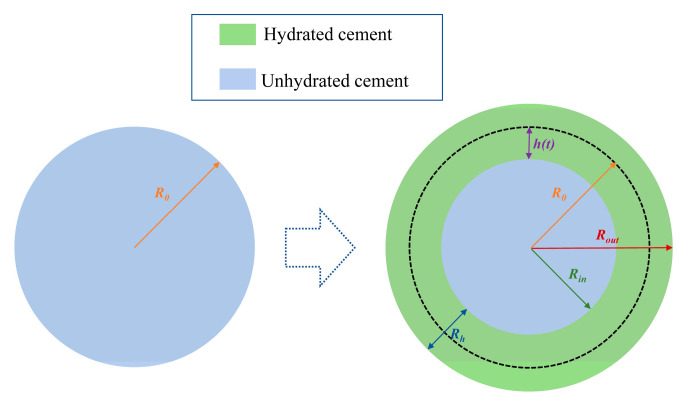
Schematic diagram of the cement hydration process.

**Figure 19:**
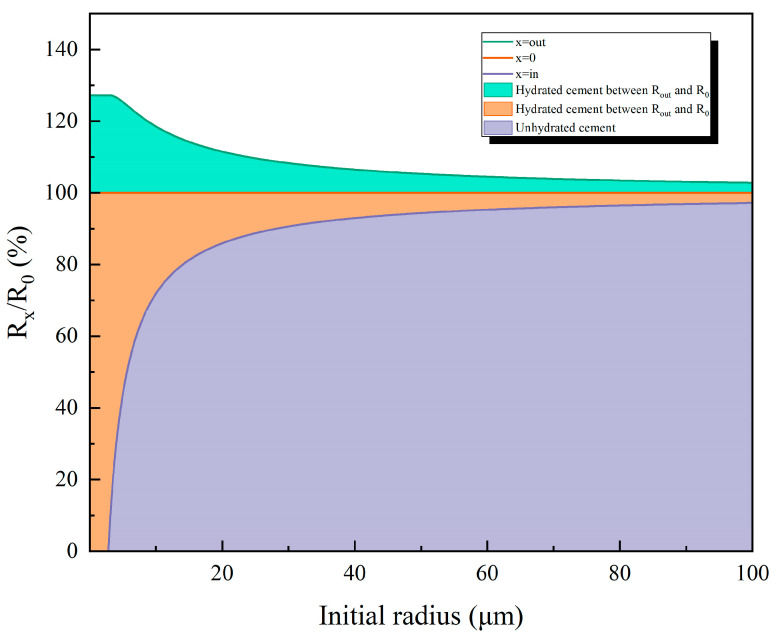
Relationship among hydration outer radius, hydration shell, and initial radius.

The outer radius of cement particles during hydration correlates with their initial particle size. Figure 18 illustrates the evolution of different “radius” parameters throughout the hydration process. At a hydration depth of 2.81 μm, we calculated the relationship among the hydration outer radius ($R_{out}$), hydration inner radius ($R_{in}$), and initial particle radius ($R_{0}$) through volumetric analysis of cement consumption and hydration product formation. This relationship is graphically presented in Figure 19. Notably, when the initial radius surpasses the hydration depth threshold, the unhydrated cement core (represented by $R_{in}$) expands rapidly due to the cubic scaling of volumetric ratios. At $R_{0}$ = 20 μm, the unhydrated portion already exceeds 80% of the original radius, after which this growth trend gradually stabilizes.

**Figure 20:**
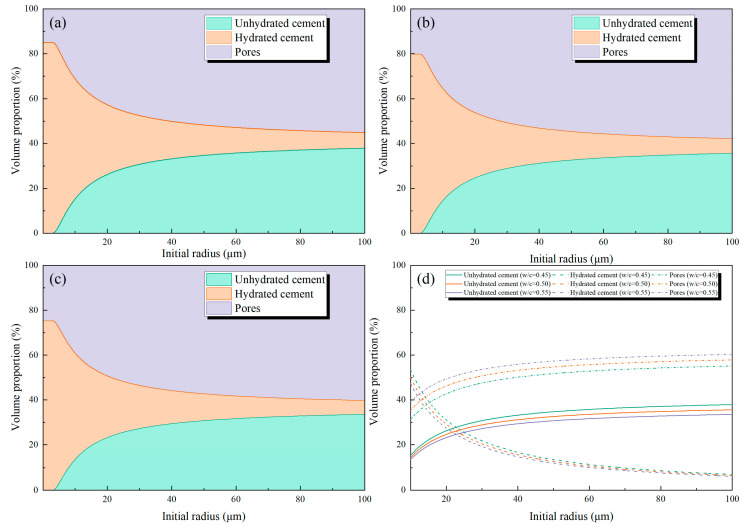
Volume proportion: (**a**) w/c = 0.45, (**b**) w/c = 50, (**c**) w/c = 0.55, and (**d**) overview.

Figure 20 shows the proportion of hydrated cement volume, unhydrated cement volume, and pore volume. The initial radius increase leads to an increase in the proportion of unhydrated cement volume, but at the same time, the proportion of pore volume increases. The former will ameliorate the self-healing ability, and the latter will deteriorate the compressive strength. From Figure 20d, it is clear that cement with a lower water/cement ratio has a larger proportion of unhydrated cement volume and a smaller proportion of pore volume than cement with a higher water/cement ratio.

**Figure 21:**
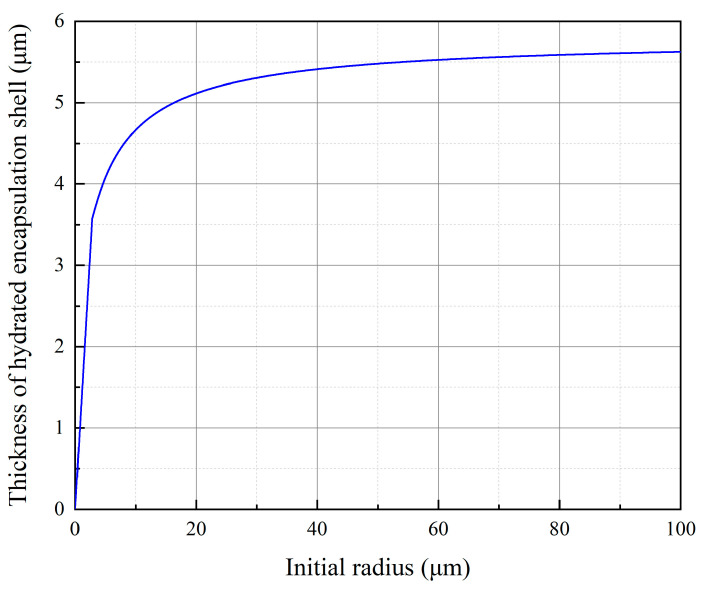
Relationship between the thickness of the hydrated encapsulation shell and the initial radius.

The relationship between the thickness of the hydrated encapsulation shell and the initial radius is shown in Figure 21. When the initial radius of the particles is greater than 20 μm (particle size of 40 μm), theoretically, “cement microcapsules” with a hydration shell layer of 5~6 μm can be formed. These “microcapsules” contain unhydrated cement inside the shell. Crack formation in cementitious materials establishes environmental connectivity through fractures, enabling hydration-driven self-healing. Coarse cement particles may develop a distinctive “hydrated shell-unhydrated core” structure due to incomplete central hydration, analogous to microcapsule technology. The hydrated outer layer demonstrates exceptional compatibility with cement matrices through interfacial integration during hydration processes. Moreover, the self-healing material itself is the product of the continuous hydration of unhydrated cement or some components in the cement and can also form an effective combination with the cement substrate after healing.

The proportion of coarse particles above 40 μm in each group can be calculated according to the size distribution of the cement, and the results are shown in Table 5. Comparing the P0-0.50 and P10-0.50 groups, the content of >40 μm particles in the P10-0.50 group is more than seven times that of the P0-0.50 group, which may explain the significant changes in the performance of these two groups.

## 4. Conclusions

Incorporating cement containing coarse particles (10–30%) enhances mortar self-healing, improving compressive strength recovery (up to 87.66% at 28 days) and ultrasonic wave velocity restoration while maintaining mechanical integrity. A lower water/cement ratio (0.45) optimizes performance, achieving 89.44% strength recovery and 21.5 MPa gains by preserving unhydrated cement for sustained hydration. By 56 days, mixes with coarse particles had exceeded 97% of the reference strength, demonstrating long-term repair.

The self-healing mechanism relies on coarse particles (>40 μm) forming microcapsule structures: hydrated shells (5–6 μm thickness) encapsulate unhydrated cores. Cracks activate these cores, enabling secondary hydration to seal damage. Optimal proportions balance strength and healing. Lower water/cement ratios reduce porosity, thereby enhancing matrix density and unhydrated cement reserves.

The optimal mix (10% coarse particles, 0.45 water/cement ratio) achieved 92.65% of the reference strength at 28 days, alongside robust self-healing. Tailoring particle size and water/cement ratios offers a pathway to durable, self-repairing cementitious materials, promising enhanced infrastructure resilience and sustainability.

The method of directly using coarse cement particles encapsulated by hydration products as microcapsules, compared to traditional microcapsule self-healing technology, does not require additional materials and complicated processes, which can reduce the energy consumption and carbon emissions generated in the preparation of additional materials and processes. Moreover, due to the increase in the content of coarse particles, the energy consumption and carbon emissions per unit volume of cement during the grinding process are also reduced. Therefore, this method is more economical and environmentally friendly.

## Figures and Tables

**Table 1:** Screening time and median diameter.

Groups	t0	t1	t2	t3	t4
Screening time/s	0	60	90	120	180
Median diameter/μm	8.25	8.28	8.56	11.67	12.79

**Table 2:** Mixing proportion and grouping of cement mortar.

Groups	Initial Cement/g	Addition of Coarse Particles/g	Sand/g	Water/g	w/c
P0-0.50	450.0	0	1350.0	225.0	0.50
P10-0.50	405.0	45.0	1350.0	225.0	0.50
P20-0.50	360.0	90.0	1350.0	225.0	0.50
P30-0.50	315.0	135.0	1350.0	225.0	0.50
P40-0.50	270.0	180.0	1350.0	225.0	0.50
P0-0.45	450.0	0	1350.0	202.5	0.45
P0-0.55	450.0	0	1350.0	247.5	0.55
P10-0.45	405.0	45.0	1350.0	202.5	0.45
P10-0.55	405.0	45.0	1350.0	247.5	0.55

**Table 3:** Compressive strength recovery rates of each group.

Groups	Compressive Strength Recovery Rate/%
P0-0.50	74.11
P10-0.50	87.66
P20-0.50	83.44
P30-0.50	82.67
P40-0.50	76.63
P0-0.45	74.85
P0-0.55	74.63
P10-0.45	89.44
P10-0.55	81.91

**Table 4:** Parameters of P.I. 42.5 cement [38].

Parameters	$K_{NG}\;(h^{-1})$	$K_{I}\;(\mu m\cdot h^{-1})$	$K_{D}\;({\mu m}^{2}\cdot h^{-1})$	n
P.I. 42.5 cement	0.0504	0.0138	0.0061	1.7510

**Table 5:** Percentage of >40 μm particles.

Groups	Percentage of >40 μm Particles
P0-0.50	0.21
P10-0.50	1.49
P20-0.50	2.77
P30-0.50	4.05
P40-0.50	5.33

## Data Availability

The original contributions presented in this study are included in the article. Further inquiries can be directed to the corresponding author.
